# Editorial: Immune profile after autologous hematopoietic stem cell transplantation for autoimmune diseases: Where do we stand? Volume II

**DOI:** 10.3389/fimmu.2022.1038338

**Published:** 2022-10-14

**Authors:** K. C. R. Malmegrim, A. Toubert, D. Farge, M. C. Oliveira

**Affiliations:** ^1^ Department of Clinical Analysis, Toxicology and Food Science, School of Pharmaceutical Sciences of Ribeirão Preto, University of São Paulo, Ribeirão Preto, Brazil; ^2^ Center for Cell-Based Therapy, Regional Hemotherapy Center of the Ribeirão Preto Medical School, University of São Paulo, Ribeirão Preto, Brazil; ^3^ Université de Paris, Institut de Recherche Saint Louis, EMiLy, Paris, France; ^4^ Institut National de la Santé et de la Recherche Médicale (INSERM) Unité Mixte de Recherche Scientifique (UMRS) 1160, Microenvironment, Lymphocyte Development and Homing, Paris, France; ^5^ Laboratoire d’Immunologie et d’Histocompatibilité, Assistance Publique-Hôpitaux de Paris (AP-HP), Hopital Saint-Louis, Paris, France; ^6^ Unité de Médecine Interne (UF 04): CRMR MATHEC, Maladies Auto-immunes et Thérapie Cellulaire. Centre de Référence des Maladies auto-immunes systémiques Rares d’Ile-de-France, Hopital Saint-Louis, Assistance Publique-Hôpitaux de Paris (AP-HP), Paris, France; ^7^ Université de Paris, Institut de Recherche Saint-Louis (IRSL), Recherche Clinique Appliquée à l'Hématologie, Paris, France; ^8^ Department of Medicine, McGill University, Montreal, QC, Canada; ^9^ Division of Rheumatology, Allergy, Immunology and Immunotherapy, Department of Internal Medicine, Ribeirão Preto Medical School, University of São Paulo, Ribeirão Preto, Brazil

**Keywords:** autoimmune disease, autologous hematopoietic stem cell transplantation, immune monitoring, multiple sclerosis, systemic sclerosis, polychondritis

The field of hematopoietic stem cell transplantation (HSCT) for autoimmune diseases has evolved over the past 25 years (1). Selection criteria and patient management strategies have been refined and associated with improvements in disease control and progression-free survival (2). Nowadays, autologous HSCT protocols are robust, reproducible, and accepted all over the world. For specific autoimmune diseases, such as multiple sclerosis and systemic sclerosis, randomized controlled trials have shown the superiority of transplants over conventional treatments (3–7), and autologous HSCT has been incorporated into treatment guidelines and recommendations (8–11).

Amongst all the other autoimmune diseases which may be considered for HSCT, including rheumatic, neurological, or inflammatory bowel diseases, and are already well-established indications for autologous HSCT, new indications and approaches have been investigated. Veldkamp et al. report the outcomes of a young patient with life-threatening juvenile relapsing polychondritis treated with HSCT. After the short-term effectiveness of autologous HSCT, long-term clinical remission was achieved after a subsequent allogeneic HSCT. Interestingly, the authors show a parallel between clinical outcomes after each of the transplants and the reconstitution of NK cells and lymphocyte subpopulations.

Investigations on how the innate and adaptative immune systems reconstitute after autologous HSCT and the identification of immune correlates of different clinical outcomes are essential to further improve the field and expand our knowledge on the approach of autoimmune diseases in general. Four articles address different aspects of the immune reconstitution after autologous HSCT for multiple sclerosis in this Research Topic. Cencioni et al. provide a very comprehensive review, starting with a discussion on how clinical outcomes of HSCT contrast with those of patients treated with the available disease-modifying therapies and then addressing many immune monitoring studies in different HSCT scenarios. The highlights of this review are the very informative figures and a rich discussion on future questions to be addressed, such as the role of self-reactive memory cells in disease reactivations after HSCT, secondary autoimmune diseases, and response to vaccines.

Massey et al. outline a novel methodology for analyzing T-cell receptor (TCR) repertoires for clones characteristic of autoimmunity. They use the bSONIA algorithm that provides a method to quantify the variability of the ‘probability of generation’ and ‘probability of survival to the periphery’ within an individual’s TCR profile. In this context, MHC class II allele HLA-DRB1*15:01 has been demonstrated to confer a three-fold increase in the risk of MS susceptibility in numerous gene association studies across diverse population groups. In a cohort of MS patients expressing the MS risk allele HLA DRB1*15:01, public clones are probed as potential biomarkers of disease. In this work, the authors perform an exploratory analysis of public clonotypes which may be relevant to MS pathogenesis, focusing on public CD4^+^CD45RO^+^ clones within HLA DRB1*15:01 positive patients as they hypothesize this would provide the greatest likelihood of defining pathogenic clones. The authors analyze the public clonotypes in the context of clinical response and demonstrated that autologous HSCT depletes public CD4^+^ memory clonotypes including those with potential autoreactivity. In addition, within the CD4^+^CD45RO^+^ pool of HLA DRB1*15:01 positive individuals, they identify two clonotypes with sequence homology (a shared GXNQPQHF motif) which were undetectable following AHSCT. The authors suggest that although the analysis is still exploratory, this methodology may prove useful for immune repertoire studies of autoimmune diseases. The authors demonstrate that autologous HSCT induces sustained periods of disease remission through dynamic changes in clonal T cell repertoire up to 36 months after transplantation.

Hendrawan et al. compare the cytokine levels in the peripheral blood of patients with multiple sclerosis or non-Hodgkins lymphoma, treated with autologous HSCT. Most pro-inflammatory cytokines were reduced in the multiple sclerosis group after HSCT compared to the hematological malignancy group. In patients that reactivated multiple sclerosis after HSCT, interleukin-17 levels increased, indicating that suppression of Th17 cytokines is essential for disease remission after autologous HSCT. These results demonstrate the importance of considering adequate control groups in future immune monitoring studies (12).

Ruder et al. address the dynamics of the innate immune system after autologous HSCT for multiple sclerosis. The authors demonstrate that HSCT induces an increase in the frequency and absolute numbers of CD56^bright^ NK cells that have immunoregulatory properties, and a decrease in CD56^dim^ NK cells, which have cytotoxic functions. In addition, they show that pro-inflammatory CD161^+^ NK cells and innate-like T-cells also decreased after HSCT. Overall, their data support an enhanced immune regulation by CD56^bright^ NK cells and the efficient reduction of proinflammatory innate-like T cells in transplanted multiple sclerosis patients, indicating that the innate immune system plays an important role in disease control after autologous HSCT.

Finally, Kawashima-Vasconcelos et al. provide an extensive review of the immune reconstitution after autologous HSCT for systemic sclerosis. The authors describe how the outcomes of the innate and adaptive immune systems correlate with clinical outcomes and disease reactivations. In this disease, the influence of the T and B-cell resetting on disease outcomes after autologous HSCT was recently well documented. The authors indicate that further cellular function assessments and comprehensive molecular analyses are needed to identify immune signatures associated with disease remission or reactivation after autologous HSCT in SSc. They propose that collaborative/multicentric approaches to evaluate the immune reconstitution of greater numbers of transplanted SSc patients worldwide may provide important answers.

Taken together, the articles from this Research Topic timely contribute to increasing the knowledge of the field. Important aspects regarding the modulation of the innate and adaptative immune system in autoimmune diseases during autologous HSCT are described and discussed. All the articles emphasize that the clinical outcomes of HSCT for autoimmune diseases have improved over the last years, but transplant protocol refinements and adjustments have reached a limit, which can only be overcome by a more detailed and deep understanding of the immune correlates of clinical outcomes. Therefore, comprehensive, systematic, multicenter, and controlled immune monitoring studies are needed and essential to enable future developments in autoimmune disease patient outcomes following autologous HSCT.

## Author contributions

All authors contributed to this publication. KM and MO wrote the draft, and AT and DF revised the final manuscript.

## Conflict of interest

The authors declare that the research was conducted in the absence of any commercial or financial relationships that could be construed as a potential conflict of interest.

## Publisher’s note

All claims expressed in this article are solely those of the authors and do not necessarily represent those of their affiliated organizations, or those of the publisher, the editors and the reviewers. Any product that may be evaluated in this article, or claim that may be made by its manufacturer, is not guaranteed or endorsed by the publisher.
